# Supplementary material for: Hypoxia-Inducible Factor-1 Alpha Expression Is Predictive of Pathological Complete Response in Patients with Breast Cancer Receiving Neoadjuvant Chemotherapy
Source: Cancers (Basel). 2022 Nov 2;14(21):5393. doi: 10.3390/cancers14215393 (PMC9656699; doi:10.3390/cancers14215393)
Supplement: Supplementary file 1 [file cancers-14-05393-s001.zip › Table S1.pdf]

Table S1. Relation between HIF-1α expression and pAKT and pMAPK

Variables	HIF-1α < 5%	HIF-1α ≥ 5%	*P*
**pAKT < 10%**	11 (50%)	11 (50%)	0.316[a]
**pAKT ≥ 10%**	34 (63%)	20 (37%)	
**pMAPK < 10%**	7 (53.8%)	6 (46.2%)	0.763[a]
**pMAPK ≥ 10%**	35 (59.3%)	24 (40.7%)	

[a] Fisher exact test
