# Supplementary material for: Hypoxia-Inducible Factor-1 Alpha Expression Is Predictive of Pathological Complete Response in Patients with Breast Cancer Receiving Neoadjuvant Chemotherapy
Source: Cancers (Basel). 2022 Nov 2;14(21):5393. doi: 10.3390/cancers14215393 (PMC9656699; doi:10.3390/cancers14215393)
Supplement: Supplementary file 1 [file cancers-14-05393-s001.zip › Table S2.pdf]

Table S2. Logistic regression model as a predictive factor of response to treatment.

| | OR | 95% CI | *P* value |
|---|---|---|---|
| **Basal phenotype** | 30.1 | 4.5-201.8 | ***0.001*** |
| **Ki-67 ≥ 20%** | 4.9 | 0.4-53.1 | 0.190 |
| **HIF-1α ≥ 5%** | 2.5 | 0.5-10.6 | 0.213 |

Abbreviations: OR, overall response
